# A randomised controlled crossover trial investigating the short-term effects of different types of vegetables on vascular and metabolic function in middle-aged and older adults with mildly elevated blood pressure: the VEgetableS for vaScular hEaLth (VESSEL) study protocol

**DOI:** 10.1186/s12937-020-00559-3

**Published:** 2020-05-12

**Authors:** Emma L. Connolly, Catherine P. Bondonno, Marc Sim, Simone Radavelli-Bagatini, Kevin D. Croft, Mary C. Boyce, Anthony P. James, Karin Clark, Reindolf Anokye, Nicola P. Bondonno, Richard J. Woodman, Amanda Devine, Seng Khee Gan, Carl J. Schultz, Richard F. Mithen, Joshua R. Lewis, Jonathan M. Hodgson, Lauren C. Blekkenhorst

**Affiliations:** 1 School of Medical and Health Sciences, Edith Cowan University, Royal Perth Hospital Research Foundation, Rear 50, Murray Street, Joondalup, Perth, WA 6000 Australia; 2 Medical School, University of Western Australia, Perth, WA Australia; 3 Centre for Integrative Metabolomics and Computational Biology, School of Science, Edith Cowan University, Joondalup, WA Australia; 4 School of Public Health, Curtin University, Perth, WA Australia; 5 Flinders Centre for Epidemiology and Biostatistics, Flinders University, Adelaide, SA Australia; 6 Liggins Institute, University of Auckland, Auckland, New Zealand; 7 Centre for Kidney Research, Children’s Hospital at Westmead, School of Public Health, Sydney Medical School, The University of Sydney, Sydney, NSW Australia

**Keywords:** Blood pressure, Cardiovascular disease, Cruciferous vegetables, Glycaemic control, Inflammation, Oxidative stress, Arterial stiffness

## Abstract

**Background:**

A diet rich in fruits and vegetables is recommended for cardiovascular health. However, the majority of Australians do not consume the recommended number of vegetable servings each day. Furthermore, intakes of vegetables considered to have the greatest cardiovascular benefit are often very low. Results from prospective observational studies indicate that a higher consumption of cruciferous vegetables (e.g. broccoli, cabbage, cauliflower) is associated with lower cardiovascular disease risk. This may be due to the presence of specific nutrients and bioactive compounds found almost exclusively, or at relatively high levels, in cruciferous vegetables. Therefore, the aim of this randomised controlled crossover trial is to determine whether regular consumption of cruciferous vegetables results in short-term improvement in measures related to cardiovascular disease risk, including ambulatory blood pressure, arterial stiffness, glycaemic control, and circulating biomarkers of oxidative stress and inflammation.

**Methods:**

Twenty-five participants (50–75 years) with mildly elevated blood pressure (systolic blood pressure 120–160 mmHg) will complete two 2-week intervention periods in random order, separated by a 2-week washout period. During the intervention period, participants will consume 4 servings (~ 300 g) of cruciferous vegetables per day as a soup (~ 500–600 mL/day). The ‘control’ soup will consist of other commonly consumed vegetables (potato, sweet potato, carrot, pumpkin). Both soups will be approximately matched for energy, protein, fat, and carbohydrate content. All measurements will be performed at the beginning and end of each intervention period.

**Discussion:**

The findings of this study will provide evidence regarding the potential cardiometabolic health benefits of cruciferous vegetables, which may contribute to the revision of dietary and clinical guidelines.

**Trial registration:**

The trial was registered with the Australian New Zealand Clinical Trial Registry on 19th September 2019 (ACTRN12619001294145).

## Background

Cardiovascular disease is the leading cause of death in Australia, accounting for 27% of all deaths in 2017 [1]. Furthermore, cardiovascular disease is the largest disease contributor to Australian health expenditure, costing the healthcare system approximately $5 billion (11%) each year [2]. One of the most important modifiable risk factors for cardiovascular disease is an unhealthy diet [3]. It is recommended that individuals consume high amounts of vegetables, fruits, mono- and polyunsaturated fatty acids, and fibre, whilst limiting their intake of total and saturated fat, cholesterol, and salt. Like cardiovascular disease, the prevalence of type 2 diabetes is increasing worldwide [4]. Adults with type 2 diabetes have a two- to three-fold increased risk of experiencing a heart attack or stroke [5]. Factors such as high blood pressure, high blood cholesterol, and overweight and obesity play a major role in the development of cardiometabolic diseases, further highlighting the importance of a healthy diet to assist in the maintenance of these factors within healthy parameters [6].

Despite knowledge of the importance of vegetables in the diet and the inclusion of such recommendations in dietary guidelines [7], only 7.5% of Australian adults consume the recommended 5–6 daily servings of vegetables (375–450 g) [8]. This is similar in many other countries [9, 10]. Inadequate vegetable intake has been attributed to 17 and 10% of the total burden of stroke and coronary heart disease, respectively [11]. In Australia, it has been estimated that total health expenditure could be reduced by $1.4 billion per year if Australians consumed the recommended 5–6 servings of vegetables daily [12].

Although current dietary guidelines recommend a high intake of all vegetables, evidence suggests that not all vegetables offer equal health benefits. In particular, studies have shown that some vegetables may be more protective against cardiovascular disease than others. Results of prospective observational studies indicate that higher consumption of vegetables from the Brassicaceae family (also called Cruciferae, known as cruciferous vegetables) is associated with lower cardiovascular disease risk [3, 13–15]. These vegetables are commonly known as cruciferous vegetables and include broccoli, cabbage, cauliflower, Brussels sprouts, kale, and bok choy. There are a number of nutrients and bioactive compounds found almost exclusively, or at relatively high levels, in cruciferous vegetables. These include, but are not limited to, organosulfur compounds, nitrate, and phylloquinone (vitamin K1). Recent studies have highlighted the potential cardiovascular health benefits of these compounds [13–18].

Extensively studied for their anti-cancer properties [15], organosulfur compounds have also been reported to have potential cardiovascular health benefits [13–15]. These compounds may slow atherosclerotic plaque progression through a reduction in inflammation and reactive oxygen species [15]. In addition to the potential benefits of organosulfur compounds found in cruciferous vegetables, nitrate has been shown to reduce blood pressure in healthy populations [19] and potentially lower the risk of cardiovascular disease clinical endpoints [16–18]. Through the enterosalivary nitrate-nitrite-nitric oxide pathway, dietary nitrate is reduced to nitrite in the oral cavity, which then enters the circulation upon swallowing and is converted to nitric oxide; a cell signalling molecule important in regulating vascular tone and integrity [20]. Currently, there is limited evidence regarding dietary phylloquinone intake and cardiovascular disease. However, some studies have suggested an inverse relationship [21, 22] and there is emerging evidence of the potential role of phylloquinone in inhibiting vascular calcification [23].

If cruciferous vegetables are indeed more superior than other vegetables in lowering risk of cardiovascular disease then targeted dietary modification including an increased intake of cruciferous vegetables has the potential to play an important role in cardiovascular disease prevention. Overall, further evidence is required to evaluate the cardiovascular health benefits of cruciferous vegetables and their bioactive constituents. Intervention studies are needed to establish the benefit of these vegetables on cardiovascular health and to determine whether cruciferous vegetables are superior to other more commonly consumed vegetable types. At present, there are no intervention trials comparing the cardiovascular health benefits of these vegetable types. The proposed study will establish the efficacy for an increase in cruciferous vegetable intake to improve measures related to cardiovascular disease risk, including blood pressure, glycaemic control, oxidative stress, and inflammation.

## Methods

### Objectives

#### Primary objective

To determine if cruciferous vegetables are superior in lowering blood pressure than more commonly consumed vegetables in middle-aged and older adults with mildly elevated blood pressure.

#### Secondary objectives

To determine if cruciferous vegetables are superior in improving arterial stiffness, glycaemic control, oxidative stress, and inflammation than more commonly consumed vegetables in middle-aged and older adults with mildly elevated blood pressure.

### Research hypotheses

The study aims to test the following hypotheses:

1. Daily consumption of cruciferous vegetables, in comparison to other commonly consumed vegetables, will lower ambulatory blood pressure in middle-aged and older adults with mildly elevated blood pressure.
2. Daily consumption of cruciferous vegetables, in comparison to other commonly consumed vegetables, will improve arterial stiffness, glycaemic control, and circulating biomarkers of oxidative stress and inflammation in middle-aged and older adults with mildly elevated blood pressure.

### Trial design

The VEgetableS for vaScular hEaLth (VESSEL) study protocol adheres to the Standard Protocol Items: Recommendations for Intervention Trials (SPIRIT) 2013 Statement (**see** Additional file 1) [24, 25]. The VESSEL study is designed as a randomised, controlled, crossover trial with two 2-week dietary intervention periods, separated with a 2-week washout period. Participants will be randomly assigned (1:1) to one of 2 sequence orders using computer generated random numbers. All participants will complete both dietary interventions. Therefore, as participants will act as their own control, this will remove any potential differences between subjects and reduce the potential bias when estimating the intervention effect. A detailed process evaluation will be performed to assess the suitability, reliability, and implementation of all procedures involved in this study (protocol in preparation).

### Participants, interventions, and outcomes

#### Study setting

This study will be conducted at the Royal Perth Hospital Research Foundation, School of Medical and Health Sciences, Edith Cowan University. The Royal Perth Hospital Research Foundation is located in Perth CBD, Australia.

#### Eligibility criteria

Participants must provide written informed consent before any physical assessments occur (see Additional file 2 for sample Participant Information and Consent Form).

##### Inclusion criteria

We will recruit ambulant community-dwelling men and women aged between 50 and 75 years who have mildly elevated blood pressure (systolic blood pressure 120–160 mmHg, inclusive, and diastolic blood pressure < 100 mmHg).

##### Exclusion criteria

Volunteers will be excluded from participation based on the following criteria: body mass index < 18.5 or ≥ 40 kg/m^2^; systolic blood pressure > 160 mmHg or < 120 mmHg; diastolic blood pressure > 100 mmHg; use of > 2 antihypertensive medications or irregular use of nitric oxide donors, organic nitrites and nitrates, and sildenafil and related drugs; diagnosed diabetes or fasting blood glucose > 6.5 mmol/L; fasting total cholesterol > 8 mmol/L; current or recent (< 12 months) smoking; adhesive allergy; regular aspirin use; medication use for thrombosis or anticoagulants (Warfarin); history of cardiovascular or peripheral vascular disease (myocardial infarction, stroke, transient ischaemic attack, amputation due to arterial insufficiency, any form of arterial revascularisation, history of exertional angina or claudication); psychiatric illness or other major illnesses, such as cancer; alcohol intake > 100 g per week; current or recent (within previous 6 months) significant weight loss or gain (> 6% of body weight) or actively trying to lose weight; pre-menopausal women; inability to attend clinic/office visits; use of antibiotics (within previous 2 months); use of antibacterial mouthwash and not willing to cease for trial duration; reported participation in night shift work during the study period; and inability or unwillingness to follow the study protocol. Volunteers with specific dietary requirements, allergies, or intolerances (e.g. following a low FODMAP diet) that will interfere with their ability to follow the dietary requirements of the study will also be excluded.

#### Recruitment

Study investigators will continue to recruit participants until an approximately equal proportion of males and females have been enrolled. The enrolment period will extend over 6 months or until adequate sample is reached.

##### Recruitment strategy

Participants will be recruited through a wide range of state and local media channels including newspaper advertisements, media articles, and radio interviews. All advertisements will have prior approval from the Edith Cowan University Human Research Ethics Committee. Responses to inquiries about participation will be answered by the study coordinator (ELC) or a research assistant using a dedicated mobile phone or email address that will be operational during business hours. Volunteers interested in participating will be sent an information pack that explains the study in detail. The study coordinator (ELC) or a research assistant will provide verbal consent from interested volunteers willing to participant to be pre-screened over the telephone. Participant will be pre-screened to determine eligibility for further physical screening at the Royal Perth Hospital Research Foundation, Perth, Australia. Physical screening will include anthropometric measurements (height and weight), blood pressure measurements, an electrocardiogram, and a fasting blood test (lipid profile and glucose test). Blood pressure will be assessed at this clinic screening visit using a CARESCAPE™ Dinamap v100 Vital Signs Monitor (GE Healthcare, Buckinghamshire, UK). Participants will be asked to rest for 5 min before 5 blood pressure and heart rate readings taken at 2-min intervals. The first measurement will be excluded and the mean of the next 4 consecutive readings will be used to determine resting blood pressure. If blood pressure is found to be outside the eligible range, volunteers will be excluded from the study and will not undergo an electrocardiogram or a fasting blood test.

#### Sample size

A sample size of 25 participants completing the trial has been calculated based on the primary outcome of mean 24-h ambulatory systolic blood pressure. To allow for up to 10% withdrawal rate, we plan to recruit 28 eligible participants to this study. We have estimated > 90% retention based on our previous studies [26]. With α = 0.05, 25 participants will provide > 90% power to detect a 2.5 mmHg difference in mean 24-h ambulatory systolic blood pressure. The power calculation is based on an SD of 14 mmHg for systolic blood pressure, a within-period correlation between systolic blood pressure measurements of 0.6, a between period correlation of the mean systolic blood pressure of 0.6, and a minimum of 40 BP measurements over each 24 h period (up to 3 measures each hour 6 am-10 pm and up to 2 each hour 10 pm-6 am) [26]. This calculation utilises the variance inflation factor (VIF) formula for a cross-over trial of VIF=N*(1-ρ)/m, where N is the number of subjects required for a parallel design, ρ is the between period correlation (0.6), and m = 2 is the number of visits per subject [27]. With α = 0.05, 25 participants will also provide > 90% power to detect a 0.30 mmol/l (~ 5%) difference in mean 2-h post-meal estimated blood glucose concentration. This is based on within-group SD of 1.5 mmol/L and a minimum of 80 glucose measures (2 meals per day with soup, for 14 days, with a measure every 15 min over 2 h) over each 2-week period, and a within-subject correlation between measures of r = 0.6.

### Assignment of interventions

#### Allocation

##### Sequence generation

Eligible participants will be randomly assigned to one of 2 sequence orders with a 1:1 allocation using computer generated random numbers.

##### Concealment mechanism

Fifty sealed opaque envelopes, numbered 1–50, each containing one of the 2 sequence orders for intervention diets will be used for randomisation by opening an envelope, in consecutive order, as participants are entered into the study. The computer-generated random numbers and sealed opaque envelopes will be completed and held by a person independent of study investigators.

##### Implementation

The study coordinator will contact the person independent of study investigators to obtain the next available envelope once an individual is deemed eligible. The envelope will be opened and the code will be recorded.

#### Blinding

Due to the nature of the interventions given, participants cannot be completely blinded to the interventions received. Participants will be blinded to which soup is the active or control. However, both soups will be different in taste and appearance. Blinding will be used for all laboratory analyses.

#### Dietary interventions

Eligible participants will complete two 2-week dietary interventions separated by a 2-week washout period where participants will return to their usual diet. During each 2-week intervention period participants will consume standard lunch and dinner meals that will be provided to standardise background diets.

The two 2-week dietary interventions will correspond to:

1. Cruciferous vegetables (**CRV**, active): 4 servings (~ 300 g) of cruciferous vegetables (broccoli, cabbage, cauliflower, and kale) per day consumed as a soup at lunch and dinner (~ 500–600 mL soup/day); and
2. other commonly consumed vegetables (**OCV**, control): 4 servings (~ 300 g) of other commonly consumed vegetables (potato, sweet potato, carrot, and pumpkin) per day consumed as a soup at lunch and dinner (~ 500–600 mL soup/day)

Four servings of vegetables (~ 300 g) daily represents an achievable daily intake of these vegetables. It is expected that participants will have a background vegetable intake of approximately 1–4 servings (~ 75–300 g) per day during the intervention periods. Therefore, the additional 4 servings of vegetables that the soups provide will shift vegetable intake to at or above the recommended 5–6 servings (375–450 g) per day.

Both active and control soups will be matched for energy (~ 600 kJ per serving). All vegetables will be boiled prior to blending into soup. A small amount of potato starch will be added to the CRV soup to approximately match the carbohydrate content with the OCV soup. There will be small differences in macronutrient content per serving: protein (CRV ~ 10 g, OCV ~ 5 g), fat (CRV ~ 0.6 g, OCV ~ 0.3 g), carbohydrate (CRV ~ 19 g, OCV ~ 27 g), and fibre (CRV ~ 9 g, OCV ~ 6 g).

Soups will be prepared at Edith Cowan University, Joondalup Campus, using standardized recipes and frozen immediately for storage.

To limit background diet variation, participants will consume frozen pre-prepared lunch and dinner meals provided by study investigators. These meals will provide approximately 1–4 servings (75–450 g) of background vegetables per day. Slight variation in vegetable intake between participants may occur, as participants will be able to select meals based on personal preference. With the exception of the intervention soups, participants will be asked to consume the same foods during both intervention periods. Participants will also be asked to replicate exactly what they ate in the 24 h prior to each clinic visit. This will allow us to directly compare the two vegetable types in both intervention periods.

Two consumer representatives provided feedback on the feasibility and palatability of the intervention soups. Consumer Advocate, Corinna Musgrave, from the Consumer and Community Health Research Network assisted in finding the most appropriate consumer representatives. Both consumers (male and female) attended the Royal Perth Hospital Research Foundation, Perth, Australia, for 1 hour. Each consumer was provided with a $35 honorarium.

##### Modifications

In the unforeseen circumstance that a study participant needs to discontinue an assigned intervention, whenever possible the participant will be retained in the trial to enable follow-up data collection and prevent missing data. Before re-commencing the assigned intervention, participants will undergo a washout period.

##### Adherence

Food and nutrient intake will be assessed daily throughout the intervention periods using dietary records. Participants will be asked to record their entire food and beverage intake each day using the application “Research Food Diary” (Xyris Software, Brisbane, Australia) or via a paper-based record. We will also use self-reported soup intake diaries and ask participants to return their empty soup containers as a measure of soup compliance.

We will measure plasma carotenoids using high performance liquid chromatography [28, 29]. Plasma carotenoids will be measured as a biomarker of vegetable intake [30]. It is expected that total plasma carotenoids will be increased substantially after both the CRV and OCV interventions. However, the carotenoid profiles will vary slightly due to the differences in individual carotenoids present in the different vegetables consumed. S-methyl cysteine sulfoxide concentration in plasma samples will be used as a biomarker of CRV intervention and will be measured using liquid chromatography-mass spectrometry (LC-MS)/MS techniques as previously described [31]. It is expected that plasma S-methyl cysteine sulfoxide concentration will be increased after CRV intervention, but not OCV intervention, and is therefore a measure of CRV intervention compliance.

##### Concomitant care

Participants will be asked to take regular medications as per usual and refrain from participating in any other research studies whilst undergoing study requirements. If a participant is a regular blood donor, the individual will be asked to postpone donating blood until after the study has been completed.

#### Participant timeline

**Fig. 1:**
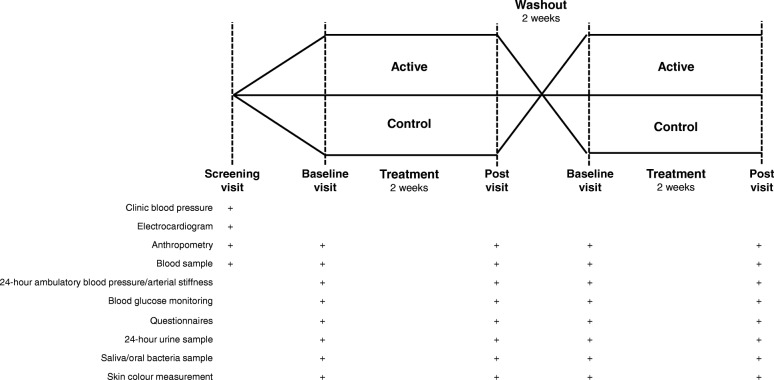
Schematic diagram of the trial design, procedures, and stages

A schematic diagram of the trial design, procedures, and stages is shown in Fig. 1. The trial consists of two 2-week intervention periods, separated by a 2-week washout period. Participants will attend 12 morning clinic visits at the Royal Perth Hospital Research Foundation over a 6 to 7-week period. Outcome assessments will be taken at the beginning and end of each 2-week intervention.

#### Outcomes

##### Primary outcome

The primary outcome of this study will be the difference between interventions (CRV, OCV) for 24-h ambulatory brachial systolic blood pressure with the additional adjustment for pre-treatment systolic blood pressure.

##### Secondary outcomes

Secondary outcomes for this study will be the difference between interventions (CRV, OCV) with the additional adjustment for pre-treatment values for the following:

- 24-h ambulatory brachial diastolic blood pressure
- 24-h ambulatory aortic systolic blood pressure
- 24-h ambulatory aortic diastolic blood pressure
- 24-h ambulatory augmentation index
- Mean continuous glucose (not adjusting for pre-treatment values)
- Oxidative stress marker (plasma F_2_-Isoprostanes)
- Inflammatory markers (serum high sensitivity interleukin-6 and high sensitivity C-reactive protein)

### Data collection, management, and analysis

#### Data collection methods

All measurements, with the exception of glucose monitoring, will be performed at the beginning and end of each 2-week intervention period. Glucose monitoring will be continuous throughout each 2-week intervention period.

##### Blood pressure

Blood pressure will be assessed as 24-h ambulatory blood pressure. Participants will be fitted with an Oscar 2™ Ambulatory Blood Pressure Monitor (ABPM) system (SunTech Medical Inc., Morrisville, NC, USA) by a trained study investigator. Whilst wearing the monitor, blood pressure (brachial and aortic) and heart rate will be measured every 20 min during the day (6 am – 10 pm) and every 30 min overnight (10 pm – 6 am). Use of the monitor will be explained by a trained study investigator, who will also instruct participants to continue daily activities and to avoid any vigorous exercise whilst wearing the monitor. Blood pressure traces that are missing more than 20% of measurements will be excluded from the analyses. If the initial tests are excluded, participants will be encouraged to repeat the test. Participants will use the same blood pressure monitor each time.

##### Arterial stiffness

Arterial stiffness will be assessed as augmentation index (AIx). AIx is a measure of systemic arterial stiffness that can be derived from aortic waveform data. This data can be obtained using the SphygmoCor component found inside the Oscar 2™ ABPM system (SunTech Medical Inc., Morrisville, NC, USA). The same algorithm as used in the SphygmoCor XCEL is used to obtain the AIx, but in an ambulatory setting instead of resting setting.

##### Glycaemic control

Participants will be fitted with a Flash Glucose Monitoring System (Freestyle Libre; Abbott Diabetes Care Inc., Alameda, CA, USA) for continuous glucose monitoring throughout the intervention periods. This system is comprised of two separate components: a Freestyle Libre sensor and reader. The sensor is small, water-resistant, and disposable, and is worn on the back of the upper arm for up to 14 days. This automatically measures glucose levels in the interstitial fluid every minute using a subcutaneous, wired, enzyme glucose sensing technology [32, 33] and stores data every 15 min for a period of 8 h. Stored glucose data is transferred to the reader by scanning the sensor. The sensor does not require calibration and allows for accurate glucose measurement without the requirement of a finger prick [34]. The screen of the reader will be covered such that participants will remain blinded to their glucose readings throughout the study.

The reader is a compact, handheld device that displays and stores up to 90 days of glucose data. After scanning the sensor, the reader displays a current glucose reading, data for the preceding 8 h (in graph form), and a trend arrow indicating if glucose is increasing, decreasing, or remaining steady. During the study, participants will be asked to scan the sensor at least once every 8 h (morning, afternoon, and evening) during both intervention periods to ensure all glucose data is captured for each 24-h period. Participants will record their diet and the timing of each meal to allow for an accurate measure of post-prandial glucose concentrations.

##### Circulating biomarkers of oxidative stress and inflammation

Fasting (≥12 h) blood samples will be collected at the beginning and end of each intervention period. F_2_-isoprostanes, a biomarker of oxidative stress, will be measured in plasma samples as total (free plus esterified) F_2_-isoprostanes using electron-capture negative-ion gas chromatography-mass spectrometry, as previously described [35]. Serum high sensitivity interleukin-6 (IL-6) and high sensitivity C-reactive protein will be measured as biomarkers of inflammation. Serum high sensitivity IL-6 will be analysed using commercially available ELISA kits, as previously described [36]. Serum high sensitivity C-reactive protein will be analysed using routine laboratory techniques at Fiona Stanley Hospital, Perth, WA. Participants will be asked to refrain from physical activity for 24-h prior to providing a blood sample.

##### Anthropometry

Anthropometric measurements will be used to assess body composition using standard protocols at each pre- and post-intervention visit. These measures will include height, weight, waist and hip circumference, and body fat mass. Participants will be wearing light clothing with no shoes or socks. Height will be measured using a wall-mounted stadiometer to the nearest 1 cm and body weight will be measured with electronic scales to the nearest 0.01 kg. A steel tape measure (W606PM; Lufkin Executive Thinline) will be used to measure waist and hip circumference. Three measurements will be taken for each site and the median of the three values will be reported. Waist circumference (cm) will be measured on bare skin at the narrowest part of the waist. In the case of the narrowest part of the waist not being visible, the waist will be measured mid-way between the lowest rib and the iliac crest. Hip circumference (cm) will be measured at the greatest lateral extension of the hips. Body fat mass will be measured using bioelectrical impedance analysis (Tanita TBF-300 Total Body Composition Analyser) and skinfold measurements. Skinfold callipers will be used to take skinfold measurements at the bicep and tricep sites.

##### Urine, blood, saliva, and oral bacteria collection

Participants will be provided with a sterilized container and instructions to collect a 24-h urine sample which will then be brought back into the clinic. Urine aliquots will be stored at − 80 °C until analysis. Urine creatinine will be measured using routine laboratory techniques at Fiona Stanley Hospital, Perth, WA.

Fasting blood samples will be collected by venepuncture. Total cholesterol, HDL cholesterol, triglycerides, and glucose will be measured using routine laboratory techniques at Fiona Stanley Hospital, Perth, WA. LDL cholesterol will be calculated using the Friedewald formula [37].

Saliva samples will be collected to assess salivary nitrate and nitrite concentrations and the nitrate to nitrite reduction ratio [38]. To obtain the saliva sample, participants will be asked to expectorate into a specimen container while sitting quietly for 5 min. Saliva aliquots will be stored at − 80 °C.

A bacterial sample will be collected from the posterior dorsal surface of the tongue using a sterile stainless-steel metal tongue cleaner. The tongue cleaner will be scraped over the dorsal surface of the tongue until a coating is visible on the tongue cleaner (~ 5 times). This coating will be removed from the tongue cleaner using a sterile collection swab and frozen at − 80 °C for later isolation of DNA.

##### Skin colour assessment

Carotenoid deposition in adipose tissue near the skin affects skin colour and has been found to correlate with fruit and vegetable consumption [39]. This will be measured as previously described [39] before and after each intervention period. Skin colour will be correlated with the Fruit and Vegetable-Specific Food Frequency Questionnaire (FVS-FFQ) and may provide a longer-term marker of fruit and vegetable consumption, and hence compliance throughout the 2-week intervention period. Eight different body sites will be measured: the sole of the foot, shoulder, right cheek, right bicep and tricep, the centre of the forehead, the back of the hand, and the palm of the hand. A Spectro-Guide 4/50 Gloss 6801 spectrophotometer with an 11 mm diameter aperture (BYK Gardner, Maryland, USA) will be used to measure the composition of the light that is reflected back from the skin after exposure to a flash of white light. This will be done three times at each session following calibration of the device according to the manufacturer’s instructions.

##### Questionnaires

Participants will be sent a link via email to complete all study questionnaires online, except for the Cancer Council of Victoria Food Frequency Questionnaire (CCV-FFQ), the FVS-FFQ, and the flavonoids questionnaire, which will be completed at each participant’s first baseline visit under the supervision of a nutritionist. At each participant’s first baseline visit, participants will complete the following questionnaires: 1) demographic and lifestyle questionnaire, a self-administered questionnaire to assess demographic characteristics such as education and previous smoking habits; 2) CCV-FFQ to determine overall habitual dietary intake, a self-administered semi-quantitative dietary questionnaire developed by the Cancer Council of Victoria, Australia [40]; 3) FVS-FFQ to determine habitual fruit and vegetable intake, an interview-based detailed questionnaire to estimate the consumption of fruits and vegetables; 4) flavonoids questionnaire, a self-administered questionnaire to estimate the consumption of foods and beverages rich in flavan-3-ols; 5) beverages questionnaire, a self-administered questionnaire used to estimate average daily/weekly intake of 12 types of beverages (tea, herbal tea, coffee, decaffeinated coffee, chocolate, fruit juice, soft drinks, diet drinks, dairy milk, non-dairy milk, fruit and/or vegetable smoothies, and water) and alcohol (beer, wine, and spirits); 6) the Community Healthy Activities Model Program for Seniors (CHAMPS), used to assess participation (activity type) and energy expenditure (intensity level) of physical activities in older adults [41]; 7) the Longitudinal Aging Study Amsterdam (LASA) questionnaire, a self-administered questionnaire to assess sedentary behaviour of older adults [42]; and 8) Perceived Stress Scale (PSS) questionnaire, a stress assessment instrument compiled of ten questions [43]. The CHAMPS, LASA, and PSS questionnaires will be administered at the beginning and end of each intervention period to monitor any changes in activity and stress levels.

Online questionnaires will be checked for completeness prior to participants coming in for their clinic visits. Participants will be asked to provide any missing information at their visit.

#### Data management

Data collection forms and protocols will be accessible to study investigators in a secured shared drive. Data for questionnaires and clinic visits will be entered electronically by the study coordinator (ELC) and checked by principal investigators (JHM and LCB). Only study investigators (ELC, JHM, LCB) will be able to access password-protected participant data and all information will remain confidential. To ensure confidentiality, data disseminated to other study investigators will be de-identified. Participant files will be locked and secured outside working hours. Data sent off-site will be de-identified to ensure confidentiality of participants.

#### Statistical methods

Statistical analyses will be performed using IBM SPSS Statistics for Windows, version 25.0 (IBM Corp., Armonk, NY, USA) and STATA, version 16.0 (Statacorp, College Station, TX, USA). Statistical significance will be set at a 2-sided Type 1 error rate of *P* < 0.05. Descriptive statistics of normally distributed continuous variables will be expressed as mean ± standard deviation (SD), non-normally distributed continuous variables as median and interquartile range, and categorical variables as number and proportion (%). The primary analyses will be performed according to a modified intention to treat protocol and will include all randomised participants for which baseline data is obtained. Multilevel models will be used to assess the effect of the dietary interventions and these will produce unbiased estimates of the intervention effects whenever there are small amounts of missing data (i.e. < 10%). Where there is > 10% missing data, multiple imputation will be used as a sensitivity analysis. Covariates will be adjusted for in analyses as necessary. Further exploratory analyses will be conducted on those participants who fully comply to intervention protocols (“per-protocol analyses”).

### Monitoring

#### Data monitoring

The study coordinator (ELC) will oversee all aspects of data monitoring with the assistance of principal investigators (JMH and LCB). Due to the scale and short duration of this trial, a data monitoring committee is not required. We also expect participants will have minimal risks undertaking this trial; therefore, no interim analyses will be conducted.

#### Harms

We expect this trial will have minimal risks to participants. However, if any solicited or spontaneously adverse events or other unintended effects of trial interventions occur, this will be reported by the study coordinator (ELC) to the principal investigators (JMH and LCB) in a prompt manner. The principal investigators (JMH and LCB) will then immediately report the event to the Edith Cowan University Human Research Ethics Committee. The outcome and actions taken will be recorded. Any serious adverse events occurring on site resulting in death will be reported immediately within 24 h and any serious AE occurring on site resulting in other circumstances other than death will be reported immediately within 72 h. All reports will be followed promptly by detailed written reports. The immediate reports and follow-up reports will identify the participant by a unique code number assigned to the trial participant rather than by the participant’s name, personal identification number, and/or addresses.

## Ethics and dissemination

### Research ethics approval

Ethics approval has been granted by the Edith Cowan University Human Research Ethics Committee (approval number: 2019–00356-BLEKKENHORST).

### Protocol amendments

Any modifications to study objectives, study design, participant population, sample size, study procedures, or significant administrative aspects will require an amendment to the protocol. Amendments will be agreed upon by study investigators and approved by the Edith Cowan University Human Research Ethics Committee prior to implementation.

### Consent or assent

Prior to the study commencing, participants will receive an information pack detailing the trial and what is expected of participation. The study coordinator (ELC) will then have an informed discussion with participants and answer any questions the individual has regarding the trial. The study coordinator (ELC) will then obtain written consent from those willing to participate in the trial. Consent will be obtained for collection and use of participant data and biological specimens in ancillary studies.

### Confidentiality

To ensure confidentiality, participants will be identified by a coded identification number. This coded identification number will be used to identify all laboratory specimens, data collection, and administrative forms to maintain participant confidentiality. All study investigators and staff will be made aware of the need for complete confidentiality when dealing with participant information. All information related to the study will be stored securely at the study site. All records that contain personal identifiers will be stored in a locked filing cabinet in a limited-access area. All databases will be secured with password-protected access systems.

### Access to data

The study coordinator (ELC) and principal investigators (JMH and LCB) will have access to the cleaned datasets. Principal investigators (JMH and LCB) will oversee intra-study data sharing. To ensure confidentiality, data shared amongst other study investigators will be de-identified.

### Ancillary and post-trial care

We expect this trial will have minimal risks to participants. However, if a serious or unexpected adverse event related to trial procedures occurs, participants will have provisional care beyond that immediately required.

### Dissemination policy

Data from this trial will not be presented in public or submitted for publication without permission of the principal investigators (JMH and LCB). On permission of principal investigators (JMH and LCB), results of this trial will be disseminated via scientific symposia and conferences and will be published in peer-review scientific journals. The requirements for authorship will adhere to scientific journal guidelines.

## Discussion

Preventative strategies are needed to mitigate the increasing prevalence of cardiovascular disease and type 2 diabetes. Diet is an important modifiable risk factor for the development of these conditions. Although the health benefits of vegetables are widely accepted, intakes of vegetables are often well below established guidelines, and intakes of vegetables linked to cardiovascular health benefits are often very limited. Therefore, the recommendation of specific types of vegetables may be warranted.

This paper outlines the protocol for a randomised controlled trial that will evaluate the impact of increasing cruciferous vegetables in a population with mildly elevated blood pressure. In this study, a comprehensive collection of vascular and metabolic outcomes will be measured to evaluate the benefit of cruciferous vegetables in comparison with more commonly consumed vegetables. The results of this trial will inform the direction of future research investigating the effect of cruciferous vegetables on cardiometabolic health outcomes and may contribute to the revision of dietary and clinical guidelines.

## Supplementary information

**Additional file 1.** SPIRIT checklist.

**Additional file 2.** Participant information and consent form.

## Data Availability

The datasets used and/or analysed during the current study are available from the corresponding author on reasonable request.
