# Supplementary material for: A randomised controlled crossover trial investigating the short-term effects of different types of vegetables on vascular and metabolic function in middle-aged and older adults with mildly elevated blood pressure: the VEgetableS for vaScular hEaLth (VESSEL) study protocol
Source: Nutr J. 2020 May 12;19:41. doi: 10.1186/s12937-020-00559-3 (PMC7218618; doi:10.1186/s12937-020-00559-3)
Supplement: Supplementary file 2 — Additional file 2. Participant information and consent form. [file 12937_2020_559_MOESM2_ESM.pdf]

Postal Address
GPO Box X2213
PERTH Western Australia 6847

Study coordinator
Tel: 0432 752 165
E-mail: thevesselstudy@ecu.edu.au

School of Medical & Health Sciences
Edith Cowan University
Royal Perth Hospital Unit
Medical Research Foundation Building
Level 4, Rear 50 Murray Street
PERTH Western Australia 6000

PARTICIPANT INFORMATION FORM

1 Introduction

This research is funded by Edith Cowan University (ECU) and the Department of Health, Western Australia.

Potential volunteers will be asked to complete a pre-screening questionnaire by telephone to establish possible eligibility. After completing the pre-screening questionnaire, if you are eligible you are invited to take part in this research project. This is because you are a man or woman aged between 50 and 75 years.

This Participant Information Form tells you about the research project. It explains the tests and treatments involved. Knowing what is involved will help you decide if you want to take part in the research. Participation in this research is voluntary.

Please read this information carefully. Ask questions about anything that you don’t understand or want to know more about. Before deciding whether or not to take part, you might want to talk about it with a relative, friend or your GP.

If you decide you want to take part in the research project, you will be asked to sign a consent form. By signing this form you are telling us that you:

- Understand what you have read
- Consent to take part in the research project
- Consent to have the tests that are described
- Consent to the use of your personal and health information as described

You will be given a copy of this Participant Information Form and the Consent Form to keep.

2 What is the purpose of this research?

Cardiovascular diseases (CVD), including heart attack and stroke, are the leading causes of death in the world. Globally, over 30% of all deaths are attributable to CVD. Suboptimal lifestyle behaviours are the leading causes of CVD globally, and most CVD-related events could be prevented or substantially delayed by increasing fruit and vegetable intakes, improving other aspects of diet, increasing physical activity and stopping smoking. Early intervention can arrest the development of CVD and prevent heart attacks and strokes. Emerging evidence suggests that some vegetables are more protective against CVD than others. Therefore, our aim is to determine whether regular consumption of certain types of vegetables results in short-term improvement in blood pressure, blood sugar levels and markers of oxidative stress and inflammation. Measurements will be performed before and after consuming soups that contain different vegetables for two 2-week periods.

3 What does participation in this research involve?

In order to determine whether you will be eligible to participate in this study, you will be pre-screened on the phone. If you are considered eligible and interested to participate, we will organise your physical screening visit (Visit 1).

Participants who are eligible and consent to participate will attend 12 study visits over 6-7 weeks. Visits will take place at the Medical Research Foundation (MRF), Royal Perth Hospital.

Prior to your first visit to MRF you will be sent an information pack, including this participant information and consent form, an appointment letter and a map of venue for the first visit.

Visit 1: The study will be explained to you in detail and any questions you have will be answered. You will be asked to sign a consent form if you wish to participate in the study. A consent form must be signed prior to any study assessments being performed. A copy of this consent form will be provided. We will determine your eligibility by measuring your blood pressure, height, and weight and performing an electrocardiogram and a blood test. We will not conduct an electrocardiogram or conduct a blood test if your blood pressure is not within the range for eligibility. Tea/coffee, toast and a spread will be provided. This visit will take approximately 60 minutes.

Visit 2: You will be asked to complete questionnaires prior to this visit and collect a 24-hour urine sample to bring along with you to your next appointment. This visit will take approximately 30 minutes.

Visit 3: Please refrain from physical activity, drinking alcohol and applying colour-tinted cream for 24-hours prior to this visit. Firstly, at this visit, you will provide your 24-hr urine sample. Your weight, waist and hip circumference, and fat mass will be measured. Following these measurements, your skin colour will be assessed as a marker of vegetable consumption. Eight different body sites will be measured: the sole of the foot (1); shoulder (2); right cheek (3); right bicep (4) and tricep (5); the centre of the forehead (6); the back of the hand (7) and palm of the hand (8). A handheld spectrophotometer will be used to measure the composition of the light that is reflected back from the skin after exposure to a flash of white light. This will be done three times. Other than gaining access to each of the body sites, the assessment of skin colour will not cause any discomfort, the flashes of light are just normal white light of a similar nature as that emitted from a light globe and do not pose any risk. After your skin colour has been assessed, you will have skinfold measurements taken at the

bicep and tricep sites using a skinfold calliper. Next, you will be asked to provide a small blood of sample (45 mL, approximately 3 tablespoons) for the measurement of markers of oxidative stress and inflammation. You will then be asked to provide a small saliva and oral bacteria sample. The oral bacteria sample will be taken by gently scraping a sterile stainless-steel metal tongue cleaner across the surface of the tongue until a coating is visible. Tea/coffee, toast and a spread will be provided. At the end of your visit, you will be fitted with a 24-hour ambulatory blood pressure monitor. This monitor will take blood pressure, heart rate and arterial stiffness measurements 3 times each hour during the day (06:00 am to 21:59 pm) and 2 times each hour during the night (22:00 pm to 05:59 am). This visit will take approximately 90 minutes.

Visit 4: At this visit, you will be fitted with a Glucose Monitoring System. This provides continuous blood sugar monitoring throughout a 14-day period. You will return your 24-hour blood pressure monitor. This visit will take approximately 5 minutes. For the next 14 days, you will be asked to consume frozen lunch and dinner meals with a soup that we will provide. At this visit, we will provide one week's worth soup and frozen meals. You will collect the rest at your next visit. You will also be asked to record all food and beverage items that you consume in the next 14 days.

Visit 5: At this visit, you will return one week's worth of empty soup containers and collect the remaining soups and frozen lunch and dinner meals. This visit will take approximately 5 minutes. You will also be provided with a urine collection jar for your next visit. Please DO NOT commence collecting your urine until 24 hours prior to your next visit.

Visit 6: This visit will be a repeat of Visit 3. This visit will take approximately 90 minutes.

Visit 7: You will return your 24-hour blood pressure monitor. Glucose data will be downloaded from your Glucose Monitoring System and the sensor will be removed. This visit will take approximately 5 minutes. For the next 14 days, you will be asked to go back to your usual diet. Please DO NOT change your diet from what you usually eat during this period. This period should reflect your diet coming into the study. You will also be provided with a urine collection jar for your next visit. Please DO NOT commence collecting your urine until 24 hours prior to your next visit.

Visits 8 to 12 will be a repeat of Visits 3 to 7.

4 Reimbursement of study costs

You can be reimbursed for any reasonable travel and parking costs associated with visiting the research centre throughout the study. This will be up to a maximum of $100 per participant for the entire study duration. This can be done by providing the study investigators with receipts for these expenses.

5 What do I have to do?

- Participation in this project requires you to consume vegetable soups along with standard frozen meals for lunch and dinner every day during two 2-week periods and to maintain your regular diet for 2 weeks in between each intervention period.
- Regular medication can be taken as usual.
- If you are a blood donor, please wait until after the study has completed to donate blood.

- This research project will require great commitment and responsibility from each participant in visits to MRF and filling in your questionnaires.

6 Do I have to take part in this research project?

- Participation in any research project is voluntary. If you do not wish to take part, you do not have to.
- If you decide to take part and later change your mind, you are free to withdraw from the study at any stage.
- If you do decide to take part, you will be given this Participant Information Form and a Participant Consent Form to sign and you will be given a copy to keep.

7 What are the possible benefits of taking part?

- At completion of the 6-week study (randomised controlled trial) we will provide you with the results of your blood tests (blood sugar and blood lipids) and blood pressure measurements.
- In addition, once all the information collected in the study has been analysed we will also provide you with a summary of the major findings of this study.
- If there are measurements taken during screening, or during the study that indicate the possibility of significantly elevated risk for heart disease, you will be advised by the research team to take these to your GP for review.

8 What are the possible risks and disadvantages of taking part?

- Having blood taken may cause some discomfort, bruising, nausea or light-headedness. If this happens, it can be easily treated. To minimise this risk a butterfly needle will be used to take blood samples and all participants will be resting in the supine position to avoid the risk of falling.
- Measurements of blood pressure may cause mild discomfort during the inflation of the blood pressure cuff around the arm. When wearing the 24-hr ambulatory blood pressure monitor, inflation of the cuff may cause minor sleep disturbance.
- Use of the sterile stainless-steel tongue cleaner may cause mild discomfort and has the potential to cause injury to the tongue. You will be instructed to use the tongue cleaner with care and to only use a gentle force when scrapping the tongue.
- Application of the glucose sensor may cause a mild stinging sensation.
- In the event of an adverse reaction to any of the procedures staff at MRF are trained in first aid.

9 What will happen to my test samples?

The collection of blood, saliva, oral bacteria and urine are mandatory components of this research project. The purpose of blood, saliva, oral bacteria and urine collection in this study is for measurements of factors related to vascular health such as blood cholesterol and blood glucose; and biomarkers of compliance with the interventions. All samples will be used for the purposes of this research and future questions related to the broader objectives of this research. Stored samples will be individually re-identifiable (coded). Privacy and confidentiality will be maintained by using password-protected databases. Samples will be

stored at the Edith Cowan University School of Medical and Health Sciences at the Royal Perth Hospital Unit, until all appropriate tests are conducted.

In addition, samples provided will be stored for future research within the Edith Cowan University School of Medical and Health Sciences at the Royal Perth Hospital Unit. The samples may only be used to investigate questions related to the broader objectives of this research. That is, research relating to the cause and prevention of vascular diseases. This may involve using samples and data collected in future studies aiming to understand factors that influence health. This can allow the investigation of research questions that require a larger number of participants than that required for this specific research study. You are asked to provide consent for the collection of your blood, saliva, oral bacteria and urine prior to the commencement of the research project.

9.1. DNA extraction and storage

We wish to seek approval to perform additional analyses on your blood samples, particularly DNA (genetic material) collection, to allow us to better understand whether you are more likely to have better health or whether your DNA may contribute to CVD-related health conditions. Please tick the corresponding box in the “Participant Consent Form” to let us know whether you give approval or not for DNA collection and analyses.

Background information

Biomarkers in your blood, including your DNA, can partly determine whether you are more likely to have better health, increased longevity and improved psychological well-being, regardless of whether or not you decide to intervene. The presence or absence of these genetic biomarkers may amount to a greater or lesser response to an intervention such as dietary and lifestyle changes. By completing additional analyses of your samples, we can determine whether certain people are more likely to respond to this type of intervention than others, due to factors such as their inherited genes.

What is DNA and how is DNA obtained from my blood sample?

DNA (deoxyribonucleic acid) is the macromolecule that genes are made up of and can be thought of as the instructions given to our cells that provide the building blocks of life. You inherit your genes from your parents, and it depends on which mix of genes you end up with as to what diseases or traits you may be at risk of developing later in life. A mutation in a gene (which may be an altered single building block of DNA or many within a gene) may be associated with health later in life.

Your blood is made up of plasma, red blood cells, white blood cells, and other molecules such as platelets, proteins and lipids. In the laboratory we will spin down your blood samples and separate out the various components into different tubes before freezing them with coded (i.e. not named) labels. We can use the part of the blood made up of white blood cells, which contain DNA, to take a snapshot of your genetic makeup.

Will my data be used in the future?

Your samples, and the data obtained, may be stored for up to 25 years for use in separate analyses in the future, but the data will remain coded to ensure you are not identifiable. After you have provided your consent, we will be able to use your samples in future studies aiming to understand factors that influence health and we will not seek further consent for their use. Privacy and confidentiality will be maintained by using password-protected databases. Blood

samples for genetic material assessment will be stored at the Edith Cowan University School of Medical, Health Sciences at Royal Perth Hospital Unit until appropriate tests are conducted, or securely kept for future analysis within 25 years of collection. However, as described below (see 'What if I withdraw from this research project?'), you may at any time withdraw your samples and any genetic material from further testing.

Dissemination of genetic information and benefits

The type of genetic data that will be generated in this study will not be useful for diagnosis of certain disease conditions. Our intention is not to determine your susceptibility to illness (e.g. we won't be able to tell you whether you have genetic predisposition to specific diseases). However, we will inform you of any results where it would be beneficial to your health and wellbeing to have this information. For example, there is a possibility that the genetic testing in this study may result in new information about health and the role of diet and exercise in specific sub-populations. If this is the case, then we will contact you with this additional information, if you wish to know it.

Agreeing to allow for further analyses, including the extraction of your DNA from your blood sample, may ultimately better the understanding of health and the response to diet and exercise interventions in the general population. There are no benefits or financial rewards for allowing for further testing or for donating your DNA to this research.

Legal Implications

We will not let anyone know that we have your DNA sample, nor will we disclose any identifiable results without your prior consent, unless disclosure is required by law. There are no commercial applications relating to this research.

10 Can I have other treatments during this research project?

Your medication and any change in your medication or intake of other supplements during the study could significantly impact on the results of this study. So it is very important to tell your research staff about any treatments or medications you may be taking, including over-the-counter medications, vitamins or herbal remedies, or other alternative treatments. You should also tell your study staff about any changes to these during your participation in the research project.

If you require an MRI, CT scan, or X-ray during the study period, please let your doctor know that you are wearing a Glucose Monitoring System sensor as this should be removed first.

11 What if I withdraw from this research project?

If you decide to withdraw from the project, please notify a member of the research team before you withdraw. If you do withdraw your consent during the research project, the principal investigator and relevant study staff will not collect additional personal information from you, although personal information already collected will be retained to ensure that the results of the research project can be measured properly and to comply with law. You should be aware that data collected by the University up to the time you withdraw will form part of the research project results. If you do not want them to do this, you must tell them before you join the research project.

12 What happens when the research project ends?

The results of the research project are intended for publication or presentation in medical literature. Once the results are determined, we will communicate the findings of the research project to you, and all other study participants. We will also provide you with a summary of values of measurements performed during the study, such as your blood pressure, blood cholesterol and blood sugar. It is a requirement of this study that you indicate via a Consent Form that you would like to learn of any information that may be significant for your health following the analysis of your results. If you require additional details on specific measurements, you can contact the investigators to request this information.

13 What will happen to information about me?

By signing the Participant Consent Form you consent to the principal investigator and relevant research staff collecting and using personal information about you for the research project. Any information obtained in connection with this research project that can identify you will remain confidential. Your information will only be used for the purpose of the research and it will only be disclosed with your permission, except as required by law. It is anticipated that the results of this research project will be published and/or presented in a variety of forums. In any publication and/or presentation, information will be provided in such a way that you cannot be identified, except with your permission.

14 Potential use of the information we collect about you in future studies?

Data collected in studies such as this are incorporated into a databank. As the databank grows, the larger number of participants included will allow investigation of research questions related to the present study: that is, the causes and prevention of heart and vascular disease. Future research projects will be either an extension of, or closely related to, this project or in the same general area of research. Any future study would include members of the current research team included in this application. Incorporation of data collected from smaller studies into larger databases is an important means of addressing research questions that cannot be addressed with small numbers of participants. Samples provided will be stored for future research within the Edith Cowan University School of Medical and Health Sciences at Royal Perth Hospital Unit.

15 Complaints and compensation

If you suffer any injuries or complications as a result of this research project, you should contact the study team as soon as possible and you will be assisted with arranging appropriate medical treatment.

16 Who has reviewed the research project?

All research in Australia involving humans is reviewed by an independent group of people called a Human Research Ethics Committee (HREC). The ethical aspects of this research project have been approved by the HREC of Edith Cowan University. This project will be carried out according to the *National Statement on Ethical Conduct in Human Research (2007).* This statement has been developed to protect the interests of people who agree to participate in human research studies.

17 Further information and who to contact

If you want any further information concerning this project or if you have any medical problems which may be related to your involvement in the project (for example, any side effects), please contact the study coordinator at thevesselstudy@ecu.edu.au.

Approval to conduct this research has been provided by Edith Cowan University, in accordance with its ethics review and approval procedures. Any person considering participation in this research project, or agreeing to participate, may raise any questions or issues with the researchers at any time.

In addition, any person not satisfied with the response of researchers may raise ethics issues or concerns, and may make any complaints about this research project by contacting the Research Ethics Officer, Edith Cowan University, 270 Joondalup Drive, JOONDALUP WA 6027 (Phone: (08) 6304 2170; Email: research.ethics@ecu.edu.au)

All research participants are entitled to retain a copy of any Participant's Information Form and Participant Consent Form relating to this research project.

Postal Address
GPO Box X2213
PERTH Western Australia 6847

Study coordinator
Tel: 0432 752 165
E-mail: thevesselstudy@ecu.edu.au

School of Medical & Health Sciences
Edith Cowan University
Royal Perth Hospital Unit
Medical Research Foundation Building
Level 4, Rear 50 Murray Street
PERTH Western Australia 6000

CONSENT TO PARTICIPATE IN THE VEGETABLES FOR VASCULAR HEALTH STUDY

I ______________________________________ have read the information provided and any questions I have asked have been answered to my satisfaction. I agree to participate in this study, realising that I may withdraw at any time without reason and without prejudice.

I consent to the storage and use of blood, saliva, oral bacteria and urine samples taken from me for use, as described in the relevant section of the Participant Information Form for:

- This specific research project
- Other research that is closely related to the objectives of this research project. This involves storage of the data and samples collected as part of this study to be used in future research relating to the cause and prevention of vascular diseases

I understand that all identifiable (attributable) information that I provide is treated as strictly confidential and will not be released by the investigator in any form that may identify me. The only exception to this principle of confidentiality is if documents are required by law.

I have been advised as to what data is being collected, the purpose for collecting the data, and what will be done with the data upon completion of the research.

I agree that any information derived from the measurements performed during the study that may be important for management of my risk of heart disease and stroke will be provided to me. I also understand that I will be informed of any measurements that indicate an elevated risk for heart disease and stroke, and will be encouraged to discuss these with my general practitioner. You can also inform study investigators whether or not you would like a nominated family member to be informed of these results.

I agree that research data gathered for the study may be published provided my name or other identifying information is not used.

I agree that my samples will be stored for up to 15 years for use in separate analyses in the future, but the data will remain coded to ensure I am not identified. I also agree that research data gathered for the study may be published provided my name or other identifying information is not used.

I provide consent for blood collection to be used for DNA testing.

( ) Yes ( ) No

Print name____

Signature ____ Date:______/_____/______
